# Preparation and Characterization of Eco-Friendly Spent Coffee/ENR50 Biocomposite in Comparison to Carbon Black

**DOI:** 10.3390/polym13162796

**Published:** 2021-08-20

**Authors:** Gunasunderi Raju, Mohammad Khalid, Mahmoud M. Shaban, Baharin Azahari

**Affiliations:** 1 School of Distance Education, Universiti Sains Malaysia, 11800 Penang, Malaysia; 2 Graphene & Advanced 2D Materials Research Group (GAMRG), School of Engineering and Technology, Sunway University, 47500 Petaling Jaya, Selangor, Malaysia; khalids@sunway.edu.my; 3 Fuel Cell Institute, Universiti Kebangsaan Malaysia, 43600 Bangi, Selangor, Malaysia; 4 Egyptian Petroleum Research Institute, Nasr City, Cairo 11727, Egypt; mahmoud_shaban26@yahoo.com; 5 School of Industrial Technology, Universiti Sains Malaysia, 11800 Penang, Malaysia; baharin@usm.my

**Keywords:** biochar, spent coffee, carbon black, epoxidized natural rubber

## Abstract

This study investigates the impact of spent coffee biochar (Biochar) compared to carbon black (CB) as a partial replacement for carbon black in epoxidized natural rubber (ENR). Particle size and elemental analysis were used to characterize the biochar and CB. Cure characteristics, tensile, thermal, and morphological properties on the effect of biochar and CB as filler were studied. It was found that incorporating 10 phr of spent coffee biochar could improve the composites’ tensile properties and thermal performance compared to carbon black. However, the addition of biochar significantly affects the maximum torque compared to CB and delays the vulcanization time. SEM study shows that biochar has a strong effect on the morphology of composite films. The FTIR graph reveals no substantial difference between compounds with biochar and CB. According to the thermal calorimetric study, the thermal stability of ENR-Biochar is higher than that of ENR-CB. Additionally, these findings suggest that the utilization of spent coffee as a sustainable biochar could be further explored, but little has been done in epoxidized natural rubber (ENR).

## 1. Introduction

Rubber composites have been investigated using renewable, nonpetroleum-based filler sources such as kenaf [1], chitosan [2], starch [3,4,5], soy proteins [6], and other fibre [7,8,9,10,11]. Even though some of these materials have shown potential reinforcing capabilities, the industry appears hesitant to experiment with filler materials that are fundamentally different from carbon black (CB) in terms of appearance and source. Global demand for petroleum products is increasing, making it vital to investigate renewable, alternative fuels and other materials to replace them if global petroleum resources run out. Carbon black is a petroleum product that dominates the rubber composite filler market due to its low cost [12]. Because of its outstanding reinforcing characteristics, low cost, and ability to be supplied in a highly pure and wide range of agglomerate sizes, carbon black, a petroleum product, has long been the standard filler for rubber composites (with the main market being the tyre industry) [13]. Biochar has recently been considered a possible replacement for carbon black additives in polymeric composites [14,15,16]. The coffee industry globally produced enormous coffee residues from manufacturing coffee beverages and instant coffee preparation, with global output estimated at 168.87 million bags (1 bag = 60 kg) in 2018–2019 [17]. Instead of being thrown away, the coffee residues could be utilized for a variety of purposes. New ideas on exploiting this residue and other renewable resources should be welcomed as a step toward a more sustainable future. Biochar is a renewable, non-petroleum-based filler substance that resembles CB in appearance. Biochar is carbon-containing charcoal generated by pyrolysis (thermal treatment in an oxygen-free atmosphere) from various carbon-containing biomass feedstocks, including hardwoods, crop residues, and poultry litter [18]. We have recently looked at using biochar as a filler in rubber composites [19,20,21,22]. ENR is a derivative of natural rubber which has two reactive sites that are unsaturated isoprene and epoxidized isoprene units. Biocomposites with good biodegradable properties can be derived from further modification of ENR [23,24,25,26].

This study aims to see if pyrolysis can be used to convert spent coffee into low-cost activated carbon. The feasibility of preparing spent coffee activated carbon by physical activation was examined in this study. The viability of natural rubber composites made from fully renewable fillers such as biochar of spent coffee (Biochar) and epoxidized natural rubber (ENR) was investigated with the intention to evaluate its potential as a sustainable and renewable substitute filler for carbon black in rubber.

## 2. Materials and Method

Epoxidized natural rubber with about 50% epoxy content (designated as ENR50) and with relative *M*w of 3.8 × 10^5^ g/mol was supplied by the Malaysian Rubber Board, Kuala Lumpur, Malaysia. The actual epoxy content was determined using Bruker Avance-400 NMR spectrometer and was found to be 51.05%. The spent coffee ground was obtained from Starbucks, Gelugor Penang. The other compounding ingredients, such as sulphur, zinc oxide, Stearic acid, sulfenamide (CBS), and carbon black N330, were purchased from Bayer Ltd. (Penang, Malaysia) and used as received. 

### 2.1. Preparation of Spent Coffee Ground and Its Derivatives

**Figure 1:**
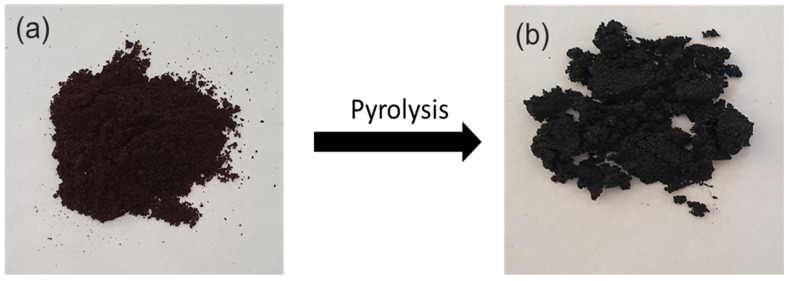
(**a**) spent coffee; (**b**) biochar.

Spent coffee grounds obtained from the local coffee shops were washed thoroughly with water to remove the impurities, dried in sunlight for two days, oven-dried at 80 °C for 24 h and labelled as SC (Figure 1a) before being stored in sealed bags. The dried SC was carbonized at 500 °C for 30 min in a furnace and cooled to room temperature once activation was complete. The samples were labelled as Biochar Figure 1b and kept in a desiccator until further use.

### 2.2. Preparation of Spent Coffee Ground and Its Derivatives—ENR Biocomposites

The preparation of ENR composite was carried out on a laboratory size 12 inches × 6 inches two-roll mill. Mixing was done in accordance with ASTM D3184-80. ENR was first masticated for 1 min, and filler was then incorporated into the mixture with continued mixing. The curing agents were then sequentially added and mixed. The compounding process was kept constant for approximately 30 min for all formulations using the formulation given in Table 1. The biocomposites obtained are designated herein as ENR-Biochar. Carbon black sample was prepared similarly, as mentioned above and designated herein as ENR-CB.

### 2.3. Chemical and Physical Material Properties 

Cure characteristics of the mixes at 150 °C were determined using a Monsanto Rheometer model MDR 2000 according to ASTM D5289-95. Then, the compounds were vulcanized with a heated compression moulding machine at 150 °C at their respective cure times.

Particle size analysis was performed on a MALVERN Zetasizer Ver. 7.11 (MAL 1029406, Germany) using light scattering on aqueous sample suspensions with a particle size detection limit of 0.6 nm–6000 nm. The measurements were performed in triplicate.

Elemental analysis of carbon (C) and hydrogen (H) was carried out using a Perkin Elmer 2400 CHNS/O series II analyzer using acetanilide as standard. Approximately 2 mg of samples were used for each measurement and was done in triplicate.

Perkin Elmer System 2000 was used to capture Fourier transform infrared spectra (FT-IR spectra with 16 scans at 4 cm^−1^ resolutions. The Attenuated Total Reflection (ATR) method was used to perform FT-IR analysis on ENR and ENR biocomposites in the wavenumber range of 4000–650 cm^−1^. X-ray diffraction (XRD) spectra were recorded using the Siemens D5000 diffractometer in step scan mode using Ni-filtered Cu K_α_ radiation (0.1542 nm wavelength). The moulded composites were scanned in transmission mode in the angle interval of 2θ = 1–12°. 

The Perkin-Elmer TGA-7 thermogravimetric analyzer was used to assess thermal weight loss. The tests were conducted using a stream of dry nitrogen gas (flow rate = 30 mL/min) at temperatures ranging from 40 to 600 °C and a heating rate of 10 °C/min. The weight of the samples utilized was between 4 and 10 mg. The FEI Quanta 650 FEG Scanning Electron Microscopy was used to examine the tensile-fractured surfaces of biocomposites. All the samples were gold coated to avoid electrostatic charging during the examination.

### 2.4. Tensile Testing 

Tensile characteristics were measured using dumbbell-shaped specimens at ambient temperature using an Instron 3366 computerized tensile tester with a 10 kN load cell, according to ASTM D412-93. The Instron machine’s crosshead speed was set at 500 mm/min.

## 3. Results and Discussion

### 3.1. Particle Size Analysis 

Table 2 shows the particle size information for various fillers made from spent coffee, biochar, and carbon black, as well as representative SEM images utilized in the analysis. The particle size of biochar after carbonization is considerably smaller than that of spent coffee agglomerates. The biochar particles have a granular, irregular—smooth, and angulated form to them. One of the most significant features in predicting the function of a particular filler in transmitting rubber characteristics is its average particle size. The higher the degree of reinforcement provided to the vulcanizate, the smaller the average particle size of a filler. Filler reinforcing effects on rubber are known to be influenced by three factors: particle size, surface activity, and particle structure [19]. Smaller particles have a greater capacity to absorb rubber molecules, increasing the filler-rubber interfaces and the performance of rubber compounds, which is consistent with our mechanical characteristics that will be discussed. The results of the elemental compositions of the CB and biochar used are presented in Table 2. Biochar contained 59.56% carbon and 6.61% hydrogen, with a 30% mass loss, whereas CB had 62.25% carbon and 0.5% hydrogen, along with other components, and a 37.25% mass loss. Pyrolysis resulted in a similar conversion of spent coffee into biochar with a carbon percentage identical to the CB. This is because the lignin content has a value on the order of spent coffee > biochar. Consequently, the study indicated that the higher the lignin level, the greater the carbon content [27]. Because of the high carbon and low ash content, the spent coffee was found to be appropriate for the preparation of carbon black.

### 3.2. Cure Characteristics and Tensile Properties

**Figure 2:**
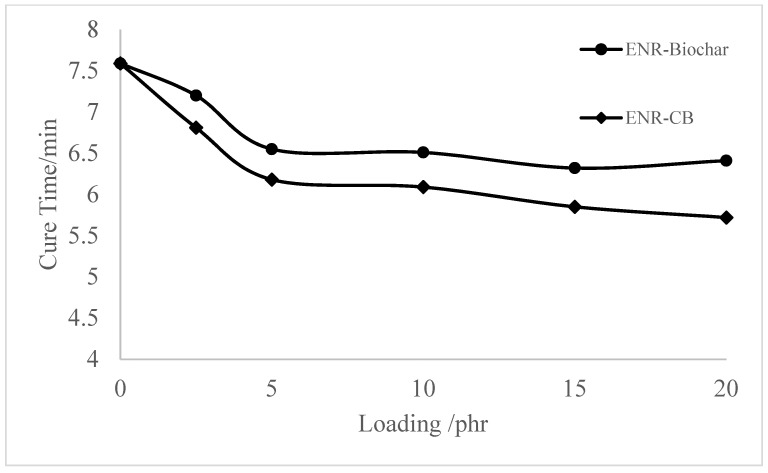
Cure time (tc90) of the ENR, ENR-Biochar and ENR-CB.

**Figure 3:**
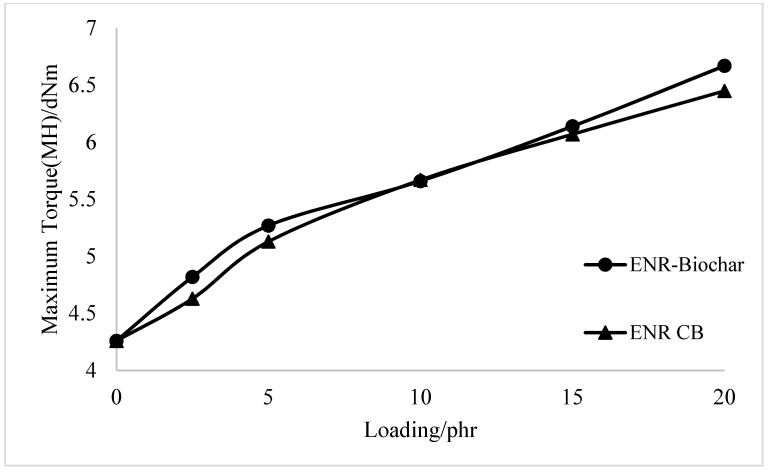
Maximum torque of the ENR, ENR-Biochar and ENR-CB.

The effect of CB and biochar in the cure characteristics of the ENR matrix is shown in Figure 2 and Figure 3, respectively. The maximum torque (MH) of the ENR filled rubber composites has steadily increased as the biochar and carbon black loading has increased. The higher MH indicates that the overall network structure, including filler networks, filler-rubber networks, and rubber networks, has increased. This is owing to the greater torque provided by the crosslink density with ENR. The tiny particle size and large specific surface area of carbon black in rubber filled with CB resulted in improved interaction between the filler and matrix, limiting the mobility of rubber molecules and resulting in higher maximum torque than activated carbon-filled rubber. As the crosslinking reaction progressed, the stiffness of rubber mixtures filled with biochar increased rapidly. The highest increase in torque gain during vulcanization was found for composites filled with biochar, which could be associated with an increased tendency for interactions [19]. The cure time (t90) of biochar and CB-filled epoxidized natural rubber compounds decreased with the increasing filler amount. The short cure time of ENR-Biochar and ENR-CB compounds could be due to the extended time on the rolling mill, as more time is needed to homogenize the compound. ENR-CB compounds demonstrated a shorter cure time than ENR- Biochar, which could be due to the smaller particle size of CB that shortens the cure time of rubber compounds [28].

**Figure 4:**
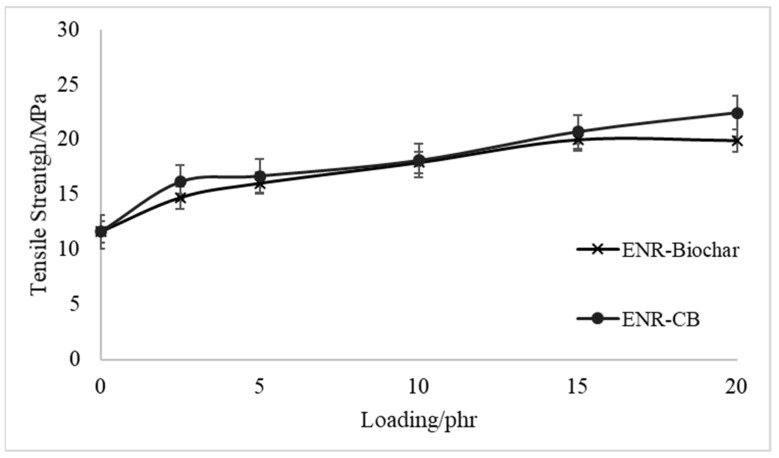
Tensile Properties of Biocomposites.

**Figure 5:**
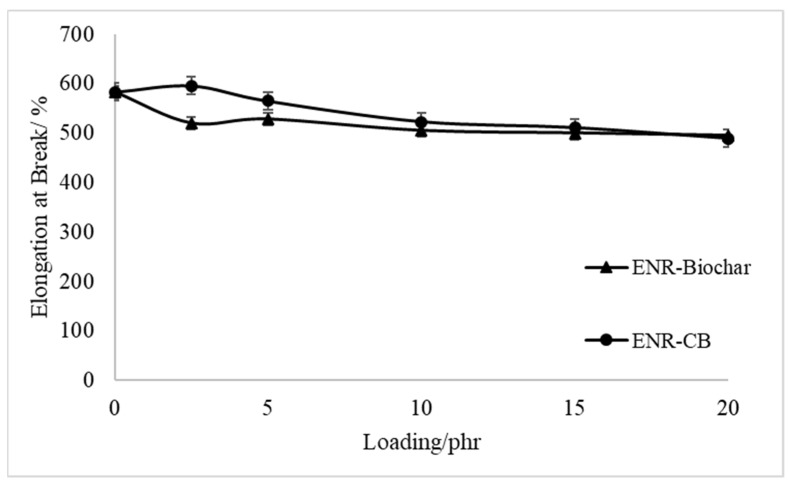
Elongation at Break of Biocomposites.

Figure 4 and Figure 5 show the tensile strength curves and elongation at the break of EBR-Biochar and ENR-CB under various loading conditions. The tensile strength properties of the biochar-filled ENR, as shown in Figure 4, increased initially and reached an optimal value when the content was at 10 phr before decreasing as the biochar loading increased. Figure 4 shows that the composites can match the tensile strength of carbon black when loaded with 10 phr of biochar. Even at 15 phr loading, the differential between biochar and CB is minimal at around 3%. This behavior could possibly be due to the chemical interactions and hydrogen bonding between the hydroxyl and epoxide groups in ENR molecules. This property creates the potential to be commercially viable for applications that need a rubbery substance. At that concentration, composites manufactured with the SC biochar fillers would be stronger and more flexible than those made with carbon black. The reinforcing impact of the fine biochar and an increase in the crosslinking density of the films might explain the first rise in tensile strength with increased biochar loading. The aggregation of biochar, which generally includes internal voids capable of absorbing polymer and partially sheltering it from stress when the rubber matrix is deformed, might explain the decrease in tensile strength seen with large biochar loadings [29]. The ENR-CB films’ tensile strength revealed that CB’s reinforcing impact was superior to the other fillers. Due to the high activity of CB and its oxidized surface, which increased with increasing degree of crosslinking of the fillers–elastomers, the improvement in tensile strength after the addition of CB lasted across the entire range studied [30]. They hypothesized that chemical and physical linkages (weak hydrogen bonds and Van der Waals forces) formed between the rubber’s carboxylic and reactive groups on the filler surfaces. However, in the epoxidized natural rubber matrix, the combination of biochar and CB decreased the elongation at break, as seen in Figure 5. The reduction is due to a significant interaction between fillers and rubber molecules, which reduced rubber vulcanizate mobility even further. Beyond 10phr loading, however, the elongation of biochar is similar to that of carbon black.

### 3.3. Fourier Transmitted Infrared (FT-IR) Analysis of ENR Biocomposites

**Figure 6:**
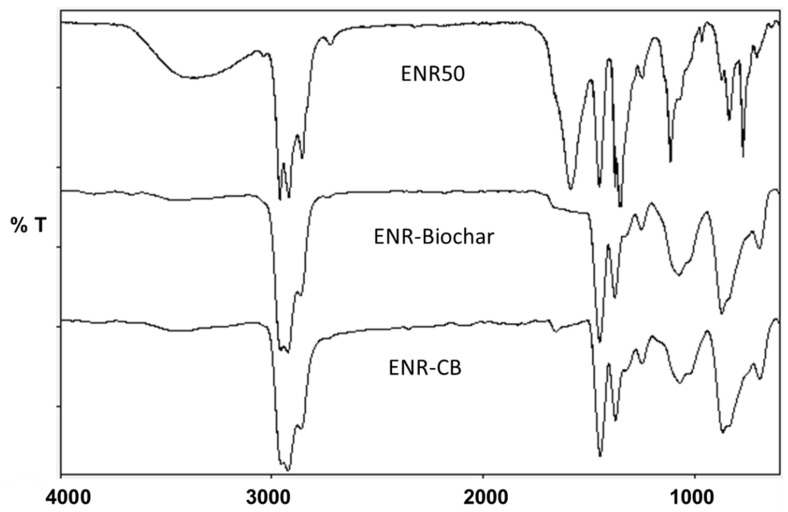
FTIR spectra of ENR 50, ENR-Biochar, ENR-CB at 10phr loading.

The FT-IR spectra of the ENR-50, ENR-Biochar and ENR-CB at 10 phr loading are shown in Figure 6. The characteristic vibrational absorption bands of ENR: 2961, 2923 and 2856 cm^−1^ are due to C–H stretchings of the –CH_3_– and –CH_2_– groups, 1660 cm^–1^ is due to the C=C stretching, 1448 and 1376 cm^–1^ are due to C–H bendings of the –CH_2_– and –CH_3_– groups, 1259 and 1018 cm^–1^ are due to C–O–C stretching and bending of the epoxide ring, 873 cm^−1^ is due to C–H bending attached to the epoxy group [31]. The locations of each frequency of the biocomposites are virtually identical to those of the ENR, as shown in the FT-IR spectra. The absorption peaks of the composite in the wavenumber range 837–1180 cm^−1^ were similarly larger than the peaks of the ENR vulcanizate spectra. This might result from intermolecular interactions between the polar groups on the biochar and the oxirane groups, or their opened rings products in ENR where there is the possibility of forming a hydrogen bond [32]. Aside from that, because biochars and CB are virtually pure carbon materials, the FTIR spectra do not have any significant functional peaks and are extremely similar.

### 3.4. X-ray Diffraction Analyses of ENR Biocomposites

**Figure 7:**
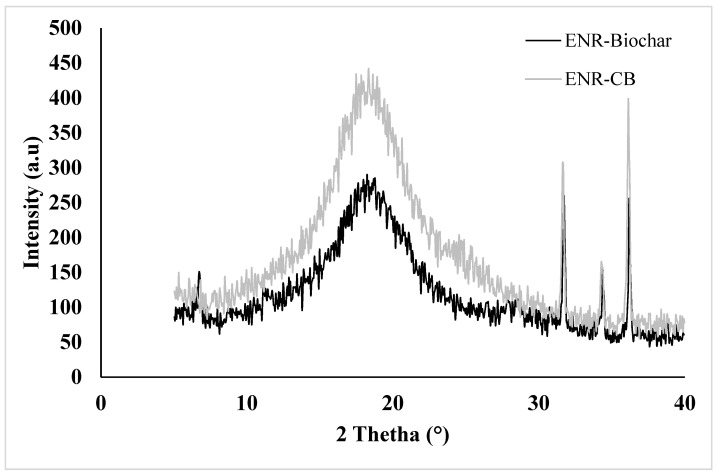
XRD patterns of ENR-Biochar and ENR-CB at 10 phr loading.

The XRD patterns of the ENR-Biochar, and ENR-CB at 10 phr loading are given in Figure 7. Due to it having the highest improvement in tensile, 10 phr loading was chosen as a test specimen for morphology study. Therefore, it is crucial to observe the filler dispersion in these samples to understand the tremendous improvement in their properties. The epoxidized natural rubber film exhibits typical amorphous polymer behaviour. A broad hump distinguishes it at an angle of 2*θ* ≈ 18°. When compared to the ENR-50, the diffraction peaks for all composites were almost identical. With increasing filler loading, the peaks related to this form of composite become stronger. This demonstrates that increasing the filler causes the composite material’s global crystallinity to rise. With an increase in the filler loading, the molecular chains of ENR will tweak the accumulated filler into a closely packed form. Diffraction patterns are created by the filler being tightly packed in the cavities of the ENR. In Figure 7, the traces for spent coffee biochar and carbon black are nearly indistinguishable, as carbon black have slightly greater intensity from 15–30° 2θ. Strong and sharp peaks confirm that their significant crystalline impurities in both biochar and carbon black. It implies that following carbonization, lignin is transformed into biochar with graphitic domains [19]. This will help strengthen its hydrophobicity and affinity for rubber and maintain the increased tensile strength.

### 3.5. Thermal Analysis of ENR Biocomposites

**Figure 8:**
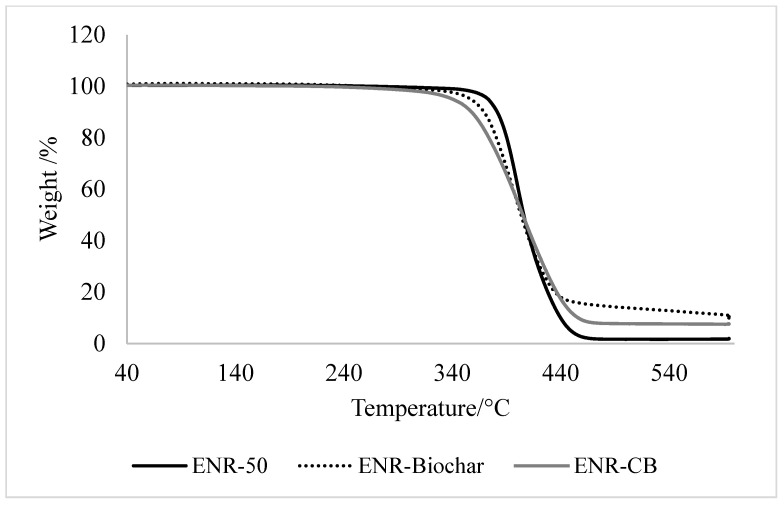
TGA of ENR vulcanizates.

In Figure 8, the weight loss versus temperature graph for ENR and ENR filled vulcanizates are presented and the thermal decomposition of ENR composites. At 600 °C, it can be observed that the gum ENR-50 had the lowest final weight. All of the composites were thermally degraded in a single step. The temperature at which biochar begins to decompose is 340 °C, while the temperature at which the greatest weight loss rate occurs is 390 °C. From the data obtained, ENR-Biochar possessed high thermal stability as it did not fully decompose upon heating up from 40–600 °C, resulting in a higher residue, proportional to the percentage of biocarbon. The remaining solid residue of biochar after heating to 600 °C was 9.8, whereas carbon black was 7.7%. This confirms that, in terms of the thermal degradation mechanism, carbon black and biochar have a similar action on the ENR rubber, and biochar composites exhibited better thermal stability.

### 3.6. Morphological Analysis of ENR Biocomposites

**Figure 9:**
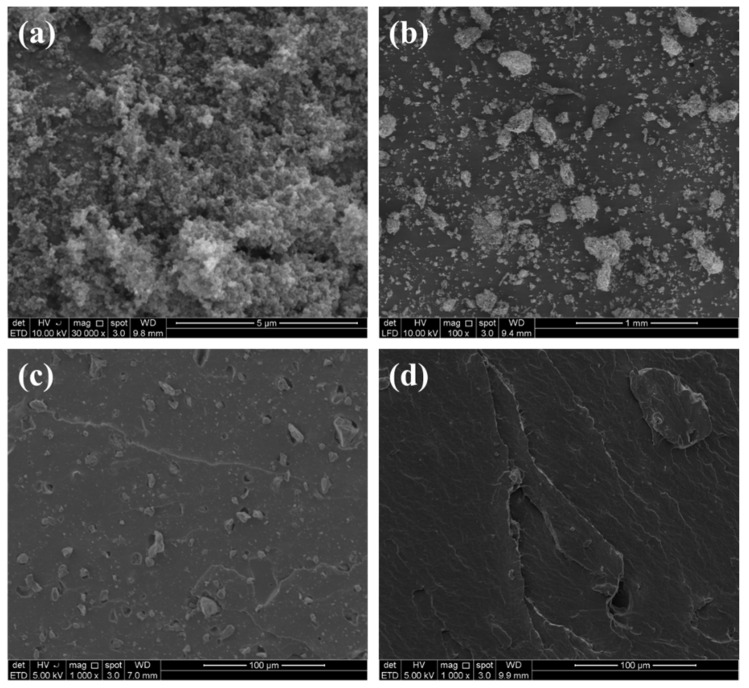
(**a**) SC (**b**) Biochar (**c**) ENR-BIOCHAR-10 phr (**d**) ENR-CB-10 phr.

As illustrated in Figure 9, scanning electron microscopy was used to compare the morphology of tensile-fractured surfaces of the composites biochar and CB. For the composites illustrated in Figure 9c, biochar particles are randomly oriented in planes parallel to the surface and are well dispersed across the whole length of the matrix. The matrix morphology in composites containing biochar shows no evidence of a phase-separated blend. The fillers are equally adhered to the surface of the rubber, as seen in the images, indicating that the process of interaction between char filler and rubber is comparable to that of conventional carbon black. The primary variations between other fillers and commercial carbon black are particle shapes, sizes, and distribution. Smaller aggregate size and more homogeneous size distribution of carbon black spheroid particles may benefit during the vulcanization process, resulting in excellent reinforcing. The carbon black seems to disperse well in the ENR due to the higher surface energy of particles of carbon black and resulted in better distribution of fillers than the biochar.

## 4. Conclusions

The mechanical and physiochemical properties of spent coffee biochar and carbon black-based epoxidized natural rubber composites were investigated in this study. Biochars made from spent coffee were able to replace 10–15% of the carbon black as it could reach almost 99% of the carbon black tensile strength with improved elongation properties. Based on the mechanical properties, spent coffee can be a partial substitute for carbon black in rubber composites, which can be a measure to reduce black carbon usage in the future. Furthermore, the thermogravimetric analysis also confirmed that the introduction of biochar to the ENR improves the thermal stability of the composites. Scanning electron microscopy further suggests that the homogeneity is greatly enhanced with the introduction of spent coffee biochar. This eventually becomes an eye-opener for the utilization of biomass as sustainable filler in the rubber industry.

## Figures and Tables

**Table 1:** Formulation of epoxidized natural rubber biocomposites.

Materials	Parts per Hundred of Rubber (phr)	
ENR50	100	100
Zinc Oxide	3	3
Stearic Acid	2	2
Biochar	0, 2.5, 5, 10, 15, 20	-
Carbon Black (N330)	-	0, 2.5, 5, 10, 15, 20
CBS	1	1
Sulfur	1.5	1.5

**Table 2:** Particle size analysis and elemental analysis results of the fillers.

Sample	Particle Size (µm)	Standard Deviation (µm)	%		
			C	H	N
SC	0.211	0.171	39.06	1.63	3.21
BioChar	0.081	0.034	59.56	6.61	2.13
CB	0.036	0.012	62.25	0.5	0.45

## Data Availability

The data presented in this study are available on request from the corresponding author.
